# Trust After Just 45 Seconds? An Experimental Vignette Study of How Leader Behavior and Emotional States Influence Immediate Trust in Strangers

**DOI:** 10.3389/fpsyg.2019.02921

**Published:** 2020-01-09

**Authors:** Olav Kjellevold Olsen, Phillip v. Heesch, Christian Søreide, Sigurd W. Hystad

**Affiliations:** ^1^Institute of Psychosocial Science, Faculty of Psychology, University of Bergen, Bergen, Norway; ^2^BI Norwegian Business School, Bergen, Norway

**Keywords:** immediate trust, democratic leadership, autocratic leadership, emotional stability, temporary groups

## Abstract

Many critical and unexpected situations are handled by people that have never met. In the literature, development of immediate trust has been identified as a prerequisite for such temporary groups and leadership to function well. Limited experimental research has studied what leadership stimulates immediate trust between strangers. The present study investigate how four leadership styles, combining autocratic or democratic leadership behavior with low or high emotional stability, is related to immediate trust in a leader displayed through a 45-s video vignette of a car accident. A sample of 280 adults, randomly assigned to one of four conditions (1, autocratic/stable; 2, autocratic/unstable; 3, democratic/stable; 4, democratic/unstable) rated immediate trust after watching the vignette. The results show that autocratic and emotionally stable leaders were on average rated higher on immediate trust than all other leadership styles, after controlling for generalized trust.

## Introduction

Trust can be defined as the willingness or intention to make oneself vulnerable to the actions of others even with limited ability to influence these actions (Mayer et al., 1995, p. 712) – and seen as a significant coordination mechanism in social cooperation and leadership. For example, trust stimulates productivity and peoples’ willingness to engage in critical situations (Colquitt et al., 2007). Conversely, lack of trust may generally impair cooperative, altruistic and extra-role behaviors in a group (Kramer and Tyler, 1996). In the literature, trust is usually seen as the outcome of shared experiences over time, providing a sufficient knowledge-basis to be able to decide whether to trust someone or not (Olsen, 2018). Accordingly, trust requires a history together, and takes time. Conversely, Meyerson et al. (1996), recognize that temporary groups, consisting of members that have never met before, still may exhibit behavior that presupposes trust, yet traditional sources of trust like familiarity, shared experience, and reciprocal disclosure are missing. Meyerson et al. (1996, p. 170) see this rapid well-functioning cooperation as a result of swift trust, seen as a rapidly arising state of trust that occurs (or not) in situations where “…people have to wade in on trust rather than wait while experience gradually shows who can be trusted and with what.” In this way, a group of strangers may successfully manage issues of vulnerability, uncertainty and expectations – and even work as an effective team.

In the literature, the time-span related to swift trust formation may variate from immediate impressions, up to several weeks of interaction (Heesch and Søreide, 2018). This is problematic because the basis for making a trust decision in seconds is probably stereotypes and schema-driven top down processing (Jarvenpaa and Leidner, 1999), quite different from that of weeks of cooperation enabling extensive testing of each other’s skills and character. Thus, in the present study, we limit the timespan for making a trust decision to only 45 s exposure, and label it “immediate trust.” Such short exposure is similar to many acute and unexpected emergencies where one must decide on whether or not to trust a stranger, for example a leader, almost immediately, with no time for familiarization beforehand. Thus, in the present study we focus on leadership in an urgent setting, encompassing time-pressure, risk of personal injury or loss of life and need of cooperation to handle the situation – combined with a need to cooperate with strangers. Such situations may require other skills and leadership approaches compared to more stable conditions with limited risk, time urgency, and familiar colleagues (cf. Kolditz, 2007).

As the awareness of immediate trust in urgent situations grows, also the search for individual and contextual predictors has increased (Blomqvist and Cook, 2018; Olsen, 2018). In the literature, variables like stereotypical impressions (Jarvenpaa and Leidner, 1999), role clarity (Curnin et al., 2015), social identity (Olsen, 2018), and generalized trust have been linked to swift trust. Furthermore, in a qualitative study of swift trust in a military operational setting, Hyllengren et al. (2011) found leadership to be the single most important factor. Few studies, however, have investigated this link further, and in particular, what leadership that best stimulates immediate trust– and under which conditions. This is thus the main scope of the present study. Given that few, if any, have studied this relationship experimentally, limiting the possibility to draw causal inferences, we will try to meet this limitation by utilizing an experimental video vignette design.

### Leadership and Immediate Trust

Hyllengren et al. (2011) found that the ability to involve and listen to the followers’ ideas and perspectives, seemingly in line with a democratic leadership orientation, stimulates swift trust in a military leader – possibly through an increased sense of control mechanism due to level of involvement and participation (Olsen, 2018). Conversely, Hannah et al. (2009) claim that people in urgent situations accept more directive and autocratic leadership. In a group of strangers, this acceptance may be particularly strong, given that no pre-established hierarchy or procedures are at place, adding to the complexity of the situation. Here, individuals that provide structure, and subsequently reduce a sense of uncertainty, may be viewed particularly positively (Olsen, 2018). This resonates with Mulder et al.’s (1986) finding that directive leaders who take control are the most effective in extreme situations. In the same vein, Sweeney (2010) found that an ability to utilize professional (tactical) skills during critical situations was the strongest predictor of trust in leaders among United States military combat personnel, far beyond the importance of variables like care, ability to involve, and friendliness, often attributed to a democratic leadership style. This is in line with Lapidot et al.’s (2007) finding that subordinates’ perception of vulnerability, which may be particularly strong among people that do not know each other in urgent situations, increases the importance of behaviors reflecting leader ability and decisiveness, compared to benevolence and participation, in order to stimulate immediate trust. Conversely, others find a more democratic leadership style better suited to stimulate swift trust between strangers. For example, a study of Scandinavian military officers found encouraging involvement and listening to followers the best leadership approach in order to promote swift trust among strangers (Hyllengren et al., 2011). Similarly, Meyerson et al. (1996) suggested that leaders should demonstrate a willingness to change plans together with the group in order to grow swift trust. Following Rotter (1990), participation may also increase a sense of internal locus of control in followers, which in turn could increase trust and willingness to be vulnerable. However, unlike the present study, these studies do not limit their investigation to urgent situations, and have a more general focus in terms of time-frame and work context, like projects and project groups, which may provide better opportunities for democratic leadership.

It is also worth noting that an individuals’ propensity to trust strangers in urgent situations may partially be an outcome of a personality trait related to trustfulness, described by McKnight et al. (1998, p.478) as generalized trust – seen as “the extent which one believes that non-specific others are trustworthy.” According to Delhey et al. (2011), this implicates that all studies investigating contextual antecedents of trust should control for this disposition.

On this basis, we suggest:

*H1*: The average trust rating for autocratic leaders is larger than for democratic leaders after 45-s exposure in an urgent situation, controlling for the influence of generalized trust of the trustor.

Furthermore, in their study of military officers, Hyllengren et al. (2011) found swift trust in leaders also highly dependent of leaders’ ability to display emotional stability. Emotional stability can be described as a tendency to arouse slowly and inhibit quickly (Eysenck and Eysenck, 1985). Thus, emotionally stable individuals are calmer and more reliable compared to neurotics who are more anxious and vulnerable. Several studies find emotional stability a predictor of leader effectiveness (Heesch and Søreide, 2018). This positive relationship may be attributed to followers’ perception of the leader as a person that will master a challenging situation, and maintain an ability to make sound judgments even during pressure, which in turn may stimulate trust, and even optimism in followers (Olsen, 2018). Such emotional stability may also be perceived as self-confidence, which Kirkpatick and Locke (1991) find one of the most influential traits on leadership effectiveness, and probably a trait that will be closely related to trust. Particularly in situations perceived as critical, where followers’ are sensitive toward qualities in a leader that may endanger vs. safeguard their own well-being and safety in the situation (Olsen, 2018).

On this basis, we propose:

*H2*: In urgent situations, immediate trust will be higher for emotionally stable leaders than emotionally unstable leaders, controlling for the influence of generalized trust.

Combining leadership behavior and emotional stability, we further propose:

*H3*: In urgent situations, leadership style and emotional stability will interact such that autocratic and emotionally stable leaders will engender higher levels of immediate trust compared with autocratic and unstable leaders, democratic and stable leaders, and democratic and unstable leaders, controlling for the influence of generalized trust.

## Materials and Methods

### Participants

Three hundred and fifty-five respondents, mostly students at the University of Bergen, participated in and completed the experiment at the basis of a convenience sampling procedure. Of this initial pool, we randomly selected 70 participants in each condition, yielding a final study-sample of 280 participants. This equalizing of cell sizes was done to circumvent the potential problems related to an unbalanced design in a factorial analysis of variance (Tabachnick and Fidell, 2014). The mean age was 26.3 (SD = 8.8) and 170 (60.7%) were female.

### Experimental Design and Procedure

The experiment was set up online. Participants first received written information about the study. After giving their informed consent, the participants completed a measure of generalized trust before watching one of four different video vignettes. The vignettes depicted a recent car accident seen from the perspective of an oncoming driver. In all videos, a first responder (i.e., the leader) meets the driver, whose behavior during the interaction with the driver constitutes the experimental conditions in this study. The experimental conditions were the following: an autocratic and emotionally stable leader (*n* = 70), an autocratic and emotionally unstable leader (*n* = 70), a democratic and emotionally stable leader (*n* = 70), and a democratic and emotionally unstable leader (*n* = 70).

After they were finished watching the videos, the participants rated the trustworthiness of the leader, using a bespoke measure of immediate trust (see section “Apparatus”). When the experiment was completed, the intent of the study was disclosed and the participants were given contact information should they have any questions.

### Apparatus

#### Video Vignettes

The video clips lasted for about 45 s and portrayed an accident with a car on fire, a possible gas leak and several persons lost in the water next to the road (available online^1^). The videos depicted scenes designed to be identical in all aspect except for leadership and emotional stability. For example, the emotional stable leader used hand gestures to reinforce verbal communication, while the unstable leader instead used non-signaling movements such as scratching the neck. We purposely filmed the scenarios at nighttime to neutralize visual biases such as attractiveness.

Three subject-matter experts evaluated the content validity of the final videos and all agreed that they exemplified the intended leadership behavior and mode of emotional stability. Pilot testing with four focus group consisting of graduate students in work and organizational psychology further showed that all but two individuals correctly classified the videos. The two mistakes turned out to be due to error on the part of the reviewer and not the video itself.

#### Immediate Trust

The Participants rated their agreement to the following three items using a 7-point Likert scale (1 = *Completely disagree* and 7 = *Completely agree*): “I trusted the person”; “I would have followed the person’s instructions”; and “I did not trust the person.” The negatively phrased item was reversed and a global index representing immediate trust was created by averaging the responses to the three items. The Cronbach’s alpha for the index was α = 0.85.

#### Generalized Trust

The respondents’ general propensity to trust strangers was measured using the single item: “Would you agree that most people in general can be trusted?,” which is based on Noelle-Neumann’s widely accepted standard question for faith in people (cf. Delhey et al., 2011). Responses were recorded using five-point Likert-type scale with anchors of 1 = *disagree* and 5 = *agree*.

### Statistical Analyses

To test our hypotheses, we performed a two-way analysis of covariance (ancova) with leadership and emotional stability as factors and generalized trust as the covariate. Three planned comparisons were conducted following a statistical significant interaction between leadership and emotional stability: The mean for autocratic and emotionally stable leaders vs. (1) the mean for autocratic/emotionally unstable leaders, (2) the mean for democratic/emotionally stable leaders, and (3) the mean for democratic/emotionally unstable leaders. A Bonferroni correction (α/3) was used to control the family wise error rate.

Partial eta-squared ($\eta_{\text{p}}^{2}$) was used to judge the magnitude of the main effects from the ancova. This effect size can be benchmarked against Cohen’s suggestions of small, medium and large effects corresponding to 0.01, 0.06, and 0.14 (cf., Richardson, 2011, p. 145). In addition, Cohen’s *d* effect sizes were computed for the three different planned comparisons. Cohen’s (1988) benchmarks were used when judging the size of the mean differences: Cohen’s *d* of 0.2, 0.5, and 0.8 are considered small, medium, and large, respectively. Because both $\eta_{\text{p}}^{2}$ and d are sample statistics, all reported values are accompanied by confidence intervals (CIs).

All analyses were performed using Stata version 15.1 (StataCorp, 2017). The d effect sizes were computed from the test statistics (*t* values) provided by the three planned contrasts, using the formulas reported by Borenstein (2009, p. 228). CIs around *d* were computed using the formula given by Hedges and Olkin (1985, p. 86).

## Results

### Experimental Conditions

A one-way ANOVA revealed that there were statistically significant differences in age between the four experimental conditions, *F*(3,276) = 3.40, *p* = 0.018. *Post hoc* comparisons using a Bonferroni correction for multiple comparisons showed that participants in the emotionally stable autocratic condition were younger (M_age_ = 23.6, SD = 7.9) than participants in the emotionally unstable autocratic condition (M_age_ = 27.9, SD = 10.8). No other pairwise comparison was statistically significant.

Overall, there were more female participants (*n* = 170, 60.7%) than male participants (*n* = 110, 39.3%) in our study. A χ^2^-test further revealed that the sex distribution was not equal in the four groups, χ^2^ (3, *N* = 280) = 11.799, *p* = 0.008. Given the overall sex distributions, there were more women than expected in the emotionally stable autocratic condition (75.7%) and fewer women than expected in the emotionally unstable democratic condition (48.6%).

### Immediate Trust

**FIGURE 1:**
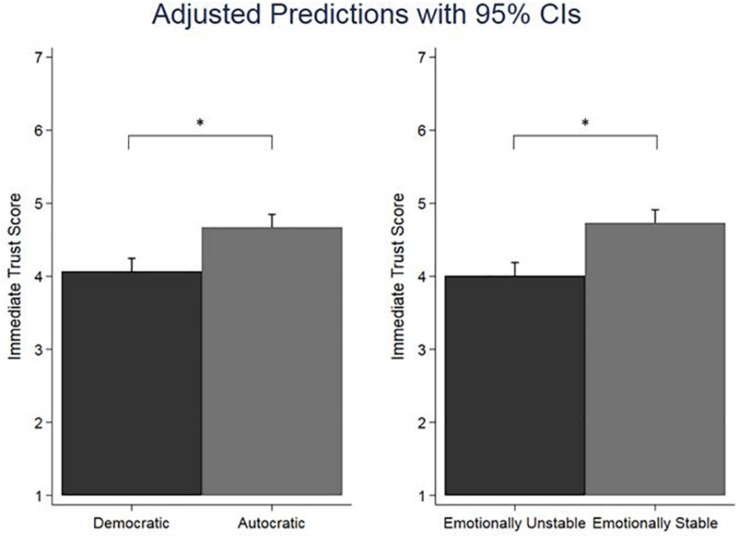
Main effects of leadership (democratic vs. autocratic) and emotional stability (emotionally unstable vs. emotionally stable) on immediate trust scores. Error bars shown are 95% confidence intervals (CIs). The asterisks indicate statistically significant differences between means (*p* < 0.001).

Given the differences in age and sex identified above, we included both variables together with generalized trust as covariates in our ancova. The results from the ancova revealed a statistical significant main effect for leadership on immediate trust ratings, *F*(1,273) = 14.99, *p* < 0.001. The partial η^2^ was 0.05 (95% CI:0.01–0.11), indicating a medium effect. The main effect of emotional stability was also statistically significant, *F*(1,273) = 27.37, *p* < 0.001, with a partial η^2^ of 0.09 (95% CI:0.04–0.16), indicating a medium effect. These two main effects are shown graphically in Figure 1, and revealed that autocratic leaders (*M* = 4.64) were on average rated significantly higher than democratic leaders (*M* = 4.02), and that emotionally stable leaders (*M* = 4.76) were on average rated significantly higher than emotionally unstable leaders (*M* = 3.89). The effect for sex was also statistically significant, *F*(1,273) = 4.93, *p* = 0.027, with an associated partial η^2^ = 0.02 (95% CI:0.00–0.06). On average, female participants (*M* = 4.48) rated the leaders higher than male participants (*M* = 4.10). Neither the effect of age, *F*(1,273) = 1.96, *p* = 0.16, nor the effect of generalized trust, *F*(1,273) = 1.52, *p* = 0.22, was statistically significant.

**FIGURE 2:**
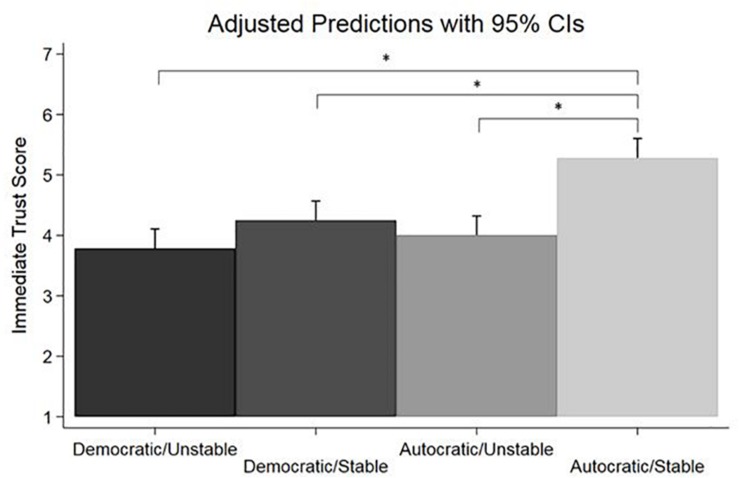
Adjusted means on immediate trust for all four experimental conditions. Error bars shown are 95% confidence intervals. The asterisks indicate statistically significant differences between means (*p* < 0.001).

The interaction between leadership and emotional stability was statistically significant, *F*(1,273) = 6.22, *p* = 0.013. The first planned contrast revealed that the emotionally stable autocratic leaders were on average rated significantly higher than their emotionally unstable counterparts, *M*_difference_ = 1.28, *t* = 5.48, *p* < 0.001, Bonferroni 95% CI: 0.71–1.84. This difference was large based on Cohen’s *d* = 0.93 (95% CI: 0.58–1.27). The second and third planned contrasts revealed that autocratic and emotionally stable leaders were on average rated significantly higher than both democratic emotionally stable (*M*_difference_ = 1.03, *t* = 4.48, *p* < 0.001, Bonferroni 95% CI: 0.48–1.58) and democratic emotionally unstable leaders (*M*_difference_ = 1.50, *t* = 6.34, *p* < 0.001, Bonferroni 95% CI: 0.93–2.06). These mean differences can both be characterized as large based on Cohen’s d of 0.76 (95% CI: 0.41–1.10) and 1.07 (95% CI: 0.72–1.42). Figure 2 shows the adjusted means for all four experimental groups.

## Discussion

The main aim of the present study was to learn more about the relationship between immediate trust and leadership during urgent situations, involving time pressure, personal risk, and need for cooperation with unfamiliar people. Notably, all hypotheses received support – and a stable emotional state combined with autocratic leadership came out as the best predictor of immediate trust. This supports previous studies, like Hannah et al. (2009) and Lapidot et al. (2007), suggesting that a directive and autocratic leader will be perceived as more competent and able to deal with an urgent situation by reducing ambiguity and stimulate a sense of control. On the other hand, this result seems to contradict Hyllengren et al.’s (2011) finding that a more democratic approach is preferable. However, it is possible that in a sample of experienced rescue workers, a more democratic leadership approach would have been preferred and seen as more trustworthy, in line with theory of situational leadership prescribing a directive approach toward less mature followers, and more delegation and involvement when working with experienced personnel (e.g., Thompson and Vecchio, 2009). Thus, a replication of the study with a more experienced sample is warranted. We would nevertheless emphasize that studies of inexperienced people, like in the present study are relevant, given that many critical situations, like car accidents, are initially managed by inexperienced bystanders. Our study therefore has good ecological validity.

As a limitation, this study has no mechanism variables included to explain the relationship between leadership and trust. Here, several variables could have been included. For example, as mediator, it may be that autocratic and stable leadership stimulates followers’ positive outcome expectations due to perceptions of leader competency and ability to cope with stressful conditions, which in turn may transfer into trust (cf. Sweeney, 2010; Olsen, 2018). Alternatively, an autocratic leadership approach may reduce perceptions of role-ambiguity and stress among the followers in an urgent situation, which may stimulate trust. This may also include moral stress, stemming from a perceived gap between a moral obligation to act rapidly, and a lack of response, which in turn may stimulate trust (Nilsson et al., 2011).

Furthermore, it should be noted that the current study does not include an assessment of trust accuracy (Schilke and Huang, 2018). The ability to decide wisely on whom to trust may represent a life or death matter in dangerous situations, and thus of high importance. Therefore, future studies should also focus on predictors of trust accuracy in these situations, possibly in terms of individual differences like level of experience, psychological hardiness or gender. Also potential perceptual biases like conformity pressure or stereotypes which may reduce accuracy of trust decisions could be relevant here. It should also be noted that emotional stability, as portrayed in the vignettes, may be interpreted as confidence more than emotional stability. Thus, in future studies, perceived level of confidence should be included as control variable.

Taken together, this study underscores the importance of acting calm and emotionally stable in order to obtain immediate trust from followers, as previously shown by Hyllengren et al. (2011). This will in itself represent an important training aim for emergency workers. Thus, another relevant line of research could investigate interventions designed to stimulate emotional stability and trust-building leadership, as well as trust accuracy to support educational programs for emergency workers like paramedics, fire departments, police and military. It is noteworthy that few contemporary programs focus on immediate trust – and trust accuracy (Olsen, 2018). This is a challenge for the future.

## Data Availability Statement

The datasets generated for this study are available on request to the corresponding author.

## Ethics Statement

The studies involving human participants were reviewed and approved by The Norwegian Data Protection Authority (DPA) and the Ethics committee of Institute of Psychosocial Science, Faculty of Psychology, University of Bergen (Norway). The patients/participants provided their written informed consent to participate in this study.

## Author Contributions

OO designed and organized data collection, theory development, preliminary analysis, and discussions. PH and CS contributed with data collection, theory development, design of vignettes, and analysis/discussion. SH contributed with theory development, design, methodology and analysis – in addition to the results.

## Conflict of Interest

The authors declare that the research was conducted in the absence of any commercial or financial relationships that could be construed as a potential conflict of interest.

## Footnotes

1 Condition 1: https://www.youtube.com/watch?v=oIPDl714DNw; Condition 2: https://www.youtube.com/watch?v=giR9sSAlW2E; Condition 3: https://www.youtube.com/watch?v=rl4LSYTYkMo; Condition 4: https://www.youtube.com/watch?v=66uEMr_Q3E4

## References

[B1] Blomqvist K. Cook K. S. (2018). “Swift trust. State-of-the-art and future research directions,” in *The Routledge Companion to Trust*, eds Searle R. Nienaber A.-M. Sitkin S. B. (London: Routledge).

[B2] Borenstein M. (2009). “Effect sizes for continuous data,” in *The Handbook of Research Synthesis and Meta Analysis*, eds Cooper H. Hedges L. V. Valentine J. C. (New York, NY: Russell Sage Foundation), 221–237.

[B3] Cohen J. (1988). *Statistical Power Analysis for the Behavioral Sciences*, 2nd Edn Hillsdale, NJ: Lawrence Erlbaum.

[B4] Colquitt J. A. Scott B. A. LePine J. A. (2007). Trust, trustworthiness, and trust propensity: a meta-analytic test of their unique relationships with risk taking and job performance. *J. Appl. Psychol.* 92 909–927. 10.1037/0021-9010.92.4.909 17638454

[B5] Curnin S. Owen C. Paton D. Trist C. Parsons D. (2015). Role clarity, swift trust and multi-agency coordination. *J. Contingencies Crisis Manag.* 23 29–35. 10.1111/1468-5973.12072

[B6] Delhey J. Newton K. Welzel C. (2011). How general is trust in “most people”? Solving the radius of trust problem. *Am. Sociol. Rev.* 76 786–807. 10.1177/0003122411420817

[B7] Eysenck H. Eysenck M. (1985). *Personality and Individual Differences: A Natural Science Perspective.* New York, NY: Springer.

[B8] Hannah S. T. Uhl-Bien M. Avolio B. J. Cavarretta F. L. (2009). A framework for examining leadership in extreme contexts. *Leadersh. Q.* 20 897–919. 10.1016/j.leaqua.2009.09.006

[B9] Hedges L. Olkin I. (1985). *Statistical Methods for Meta-Analysis.* New York, NY: Academic Press.

[B10] Heesch P. V. Søreide C. (2018). *Cooperation in the Heat of the Moment: The Effect of Leadership Behavior on Swift Trust.* Master thesis, University of Bergen, Bergen.

[B11] Hyllengren P. Larsson G. Fors M. Sjöberg M. Eid J. Kjellevold Olsen O. (2011). Swift trust in leaders in temporary military groups. *Team Perform. Manag.* 17 354–368. 10.1108/13527591111182625

[B12] Jarvenpaa S. L. Leidner D. E. (1999). Communication and trust in global virtual teams. *Org. Sci.* 10 791–815. 10.1287/orsc.10.6.791

[B13] Kirkpatick S. A. Locke E. A. (1991). Leadership: do traits matter? *Executive* 5 48–60. 10.5465/ame.1991.4274679

[B14] Kolditz T. (2007). *In Extremis Leadership: Leading As If Your Life Depended On It.* San Francisco: Jossey-Bass.

[B15] Kramer R. Tyler T. (1996). *Trust in Organizations: Frontiers of Theory and Research.* Thousand Oaks, CA: Sage Publications.

[B16] Lapidot Y. Kark R. Shamir B. (2007). The impact of situational vulnerability on the development and erosion of followers’ trust in their leader. *Leadersh. Q.* 18 16–34. 10.1016/j.leaqua.2006.11.004

[B17] Mayer R. C. Davis J. H. Schoorman F. D. (1995). An integrative model of organizational trust. *Acad. Manag. Rev.* 20 709–734. 10.5465/amr.1995.9508080335

[B18] McKnight D. H. Cummings L. L. Chervany N. L. (1998). Initial trust formation in new organizational rela- tionships. *Acad. Manag. Rev.* 23 473–490. 10.5465/amr.1998.926622

[B19] Meyerson D. Weick K. E. Kramer R. M. (1996). “Swift trust and temporary groups,” in *Trust in Organizations: Frontiers of Theory and Research*, eds Kramer R. Tyler T. (London, UK: Sage Publication Ltd), 166–195. 10.4135/9781452243610.n9

[B20] Mulder M. de Jong R. D. Koppelaar L. Verhage J. (1986). Power, situation, and leaders’ effectiveness: an organizational field study. *J. Appl. Psychol.* 71 566–570. 10.1037//0021-9010.71.4.566

[B21] Nilsson S. Sjöberg M. Kallenberg K. Larsson G. (2011). Moral stress in international humanitarian aid and rescue operations: a grounded theory study. *Ethics Behav.* 21 49–68. 10.1080/10508422.2011.537570

[B22] Olsen O. K. (2018). “Effective cooperation between strangers in unexpected and dangerous situations – a matter of “Swift Trust”,” in *Interaction:‘Samhandling’ Under Risk. A Step Ahead of the Unforeseen*, ed. Torgersen G.-E. (Oslo: Cappelen Damm Akademisk), 399–412. 10.23865/noasp.36.ch21

[B23] Richardson J. T. E. (2011). Eta squared and partial eta squared as measures of effect size in educational research. *Educ. Res. Rev.* 6 135–147. 10.1016/j.edurev.2010.12.001

[B24] Rotter J. B. (1990). Internal versus external control of reinforcement: a case history of a variable. *Am. Psychol.* 45 489–493. 10.1037//0003-066x.45.4.489

[B25] Schilke O. Huang L. (2018). Worthy of swift trust? How brief interpersonal contact affects trust accuracy. *J. Appl. Psychol.* 103 1181–1197. 10.1037/apl0000321 29963894

[B26] StataCorp (2017). *Stata Statistical Software: Release 15.* College Station, TX: StataCorp LLC.

[B27] Sweeney P. J. (2010). Do soldiers reevaluate trust in their leaders prior to combat operations? *J. Milit. Psychol.* 22(Suppl.1), 70–88.

[B28] Tabachnick B. G. Fidell L. S. (2014). *Using Multivariate Statistics (6th ed; international edition)*. Harlow, UK: Pearson Education Ltd.

[B29] Thompson G. Vecchio R. P. (2009). Situational leadership theory: a test of three versions. *Leadersh. Q.* 20 837–848. 10.1016/j.leaqua.2009.06.014

